# Transferable Plasmonic Arrays Enabling Strong Coupling with Layered Perovskites in an Active Diode Architecture

**DOI:** 10.1021/acs.nanolett.5c04569

**Published:** 2025-12-31

**Authors:** Fabien Dorey, Jonas D. Ziegler, Antti J. Moilanen, Oleh Hordiichuk, Gabriel Nagamine, Takashi Taniguchi, Kenji Watanabe, David J. Norris, Maksym V. Kovalenko, Gabriele Rainò, Lukas Novotny

**Affiliations:** † Photonics Laboratory, 27219 ETH Zürich, CH-8093 Zürich, Switzerland; ‡ Laboratory of Inorganic Chemistry, Department of Chemistry and Applied Biosciences, Institute of Inorganic Chemistry, ETH Zürich, CH-8093 Zürich, Switzerland; ¶ Optical Materials Engineering Laboratory, Department of Mechanical and Process Engineering, ETH Zürich, CH-8092 Zürich, Switzerland; § International Center for Materials Nanoarchitectonics, 52747 National Institute for Materials Science, Tsukuba, Ibaraki 305-004, Japan; ∥ Research Center for Functional Materials, National Institute for Materials Science, Tsukuba, Ibaraki 305-004, Japan; ⊥ Department of Physics and Mathematics, Faculty of Science, Forestry and Technology, University of Eastern Finland, Joensuu FI-80101, Finland; # Laboratory for Thin Films and Photovoltaics, Empa - Swiss Federal Laboratories for Materials Science and Technology, CH-8600 Dübendorf, Switzerland

**Keywords:** Surface lattice resonances, 2D materials, Strong coupling, perovskites, Light emitting devices, Polaritons

## Abstract

Plasmonic cavities provide a powerful platform for nanoscale light confinement far beyond the diffraction limit, enabling exceptionally strong light-matter interactions. Recent advances in plasmonic architectures and active materials enable stable, scalable routes toward fully integrated on-chip photonics. In this work, we demonstrate the strong coupling of 2D layered perovskites with a transferable plasmonic cavity directly fabricated on top of hexagonal boron nitride (hBN). Under optical excitation, we observe clear interaction between the exciton and cavity modes leading to strong and even ultrastrong coupling, with coupling factors *g* up to 10% of the resonance energy. Combined with the polariton mode hybridization and a waveguiding effect, this results in pronounced line width narrowing of the polariton modes well below both the bare cavity and exciton line widths. Electrical excitation of the hybrid modes is achieved via electron tunneling and simultaneous energy transfer, making this design an attractive configuration for on-chip integration.

## Graphical abstract

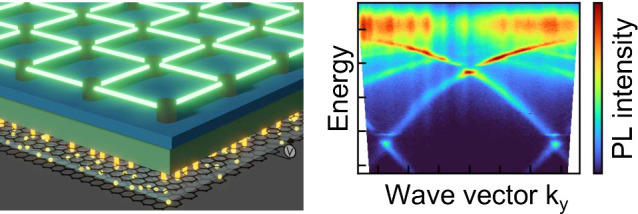

Controlling light-matter interactions at the nanoscale is a central challenge in modern photonics, with implications ranging from fundamental quantum optics to next-generation integrated photonic and optoelectronic devices.^1–4^ In particular, the regime of strong light–matter coupling—where photons and excitations such as excitons hybridize to form new quasiparticles—offers powerful tools for modifying and enhancing the optical, electronic, and even chemical properties of materials.^5,6^ This regime enables phenomena such as enhanced nonlinearities,^7,8^ long-range coherent energy transport,^9,10^ and the emergence of exotic many-body states of light,^11,12^ while also providing new platforms for scalable, actively tunable nanophotonic devices.^13,14^

A promising type of cavity to facilitate enhanced light-matter interactions are plasmonic nanoparticle arrays.^15–17^ These cavities are made of periodically spaced plasmonic nanoparticles such as gold, silver, or aluminum. Due to the regular spacing between the nanoparticles, diffracted orders of light can be guided into the cavity plane and hybridize with the localized surface plasmon resonance of the particles giving rise to surface lattice resonances (SLRs). SLRs combine plasmonic properties such as high field enhancement due to the strong field confinement at the particle sites but with reduced losses compared to other plasmonic cavities because of their photonic component.^15–17^ Indeed, line widths down to a few nanometers have been reported in the visible range and even below 1 nm in the near-infrared.^18,19^

The open cavity design is ideal for the integration of solution-based active materials, such as quantum dots or dye molecules.^16^ Here, the recent years saw both breakthrough developments in understanding polariton physics such as condensation as well as promising device architectures achieving lasing.^20–22^ For on-chip photonics, solid-state materials can offer crucial advantages in terms of electrical excitation and stability.^23^ Here, two-dimensional (2D) materials are one of the most promising material systems. 2D materials typically consist of strongly bound layers in one plane which are held together weakly by van der Waals forces in the out-of-plane direction.^24^ They offer natural quantum confinement in one direction and benefit from strongly reduced screening from the environment, leading to strongly bound excitations and high light-matter interaction.^25–27^ Furthermore, most 2D materials are easily stacked together without dangling bonds or lattice mismatch limitations common in most bulk materials.^28^

Importantly, many excitation schemes can be readily employed for layered materials, while novel charge injection methods based on 2D materials such as hBN and graphene evolve quickly.^29–31^ One of the most promising 2D materials for light-emitting devices are layered hybrid perovskites.^32,33^ This special class of 2D materials consists of alternating layers of inorganic lead-halide perovskites separated by organic spacer molecules.^34–37^ This structure not only enhances their stability compared to their 3D counterparts but also their optical properties due to the additional confinement to a 2D layer independent of the material thickness.^38^ Finally, their cheap solution-based fabrication and chemical design flexibility make them of particular interest. For example, changing the thickness of the inorganic layer or composition of the ligand has been shown to strongly change their emission wavelength and oscillator strength.^39^

In this work, we present the integration of layered perovskites and atomically thin tunnel junctions into nanoparticle arrays. Upon optical excitation, we observe a pronounced avoided crossing with coupling energies up to 270 meV, indicative of ultrastrong coupling, accompanied by line width narrowing. Furthermore, using electron tunneling in combination with energy transfer we demonstrate electrical excitation of hybrid perovskite-resonator modes.

We employ a flexible fabrication process to create transferable nanoparticle arrays on thin layers of hBN, as depicted schematically in Figure 1a. Similar to other transferable nanoparticle array approaches,^40–42^ this process allows the integration of sensitive materials such as organics or hybrid organic–inorganic perovskites into plasmonic cavities, while our method preserves atomically flat interfaces crucial for van der Waals materials. The two-step process separates the electron-beam lithography (EBL) step from the rest of the fabrication, avoiding direct exposure of the active material to the electron beam or other cleanroom fabrication steps.^43,44^

**Figure 1:**
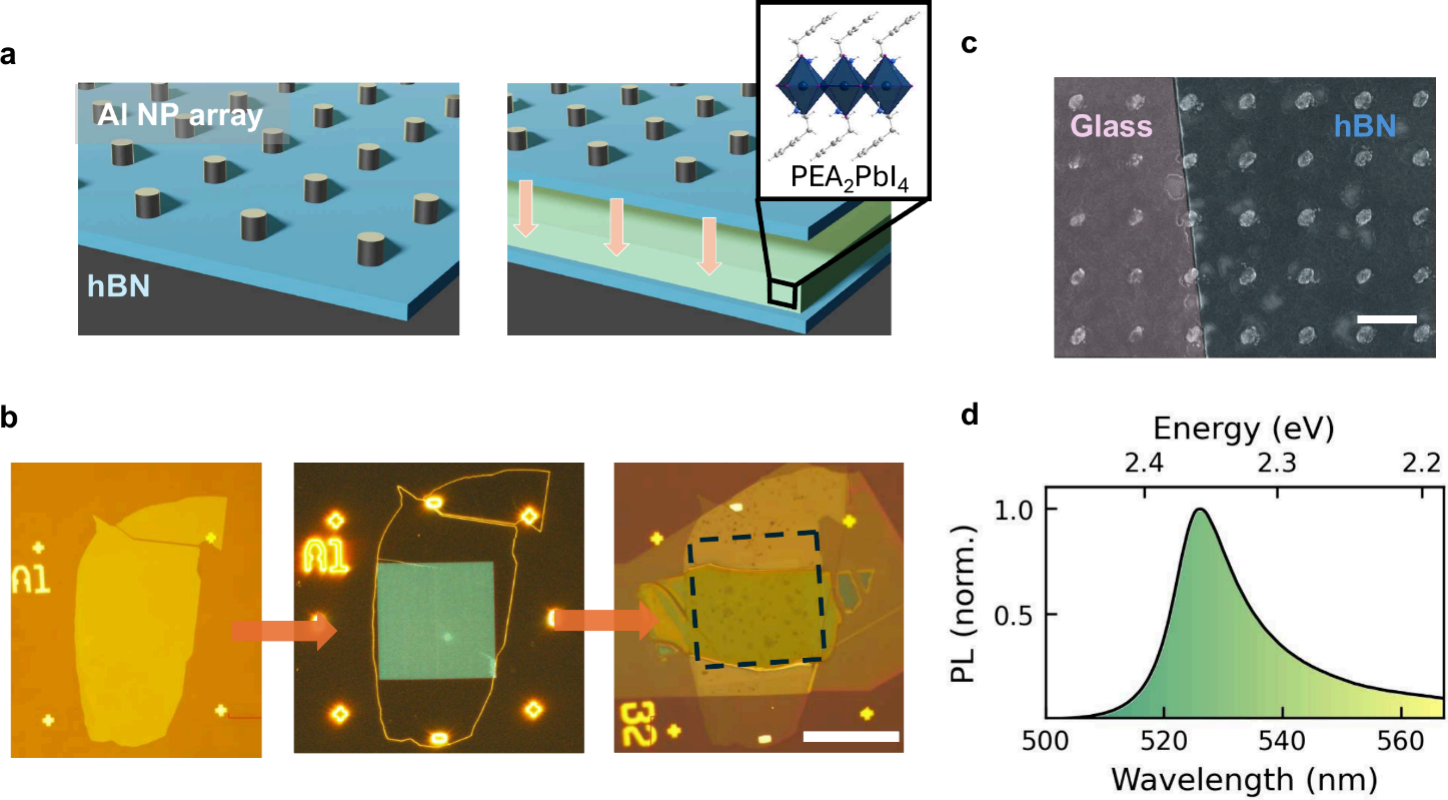
Integration of 2D perovskites with nanoparticle arrays. a) Schematic of the transferable nanoparticle array with and without 2D perovskite. The inset shows the perovskite crystal structure and its surrounding ligands. b) Left: Optical microscope image of the bare hBN flake. Center: Dark-field microscope image of a plasmonic nanoparticle array fabricated on the same hBN flake. Right: Optical microscope image of the same cavity after transfer on a 2D perovskite flake (scale bar: 50 μm). c) Scanning electron microscope (SEM) image of aluminum nanoparticles deposited on glass and hBN (scale bar: 400 nm). d) Photoluminescence (PL) spectrum of a bare *n* = 1 2D perovskite layer.

We use hybrid inorganic–organic perovskites, which combine high quantum efficiencies with narrow line width and a tunable emission range by changing their chemical composition or by varying the inorganic layer thickness (*n*-value).^39^ Similar to traditional III–V semiconductor systems, the confining potential in layered perovskites can be precisely tuned by varying the thickness of the inorganic layers. This tunability enables control over excitonic energies and optical properties, offering a versatile platform for tailored light-matter interactions and next-generation optoelectronic applications. The additional confinement from the layered structure leads to strongly bound excitons with high oscillator strength, facilitating the formation of exciton-polaritons when coupled to cavities.^45^ Furthermore, large flakes on the order of tens to hundreds of micrometers can be easily exfoliated, which fits state-of-the-art nanoparticle arrays lateral dimensions.^18^

For the fabrication, large and thin (<30 nm) hBN flakes were obtained using a polymer-based exfoliation process. Using standard EBL techniques, square arrays of nanoparticles were patterned in a poly­(methyl methacrylate) (PMMA) resist, spin coated on the glass substrate with hBN flakes. Aluminum was deposited at high rates of 5 nm/s to limit oxidization during the deposition process.^46^ We characterize the nanoparticles using optical microscopy in Figure 1b (left) and scanning electron beam microscopy (SEM) in Figure 1c. We find that except for a scaling factor of the nanoparticle sizes the deposition parameters and quality remained the same on the hBN flakes. The flakes with the nanoparticles can subsequently be transferred onto other 2D materials, using dry, polymer-based transfer method.^47^ Figure 1b (right) shows the cavity after transfer on a 2D organic–inorganic perovskite flake.

In this report, we investigate *n* = 1 perovskites (PEA_2_PbI_4_, PEA = phenylethylammonium) which show a narrow emission at 2.36 eV; see Figure 1d. One of the main drawbacks of perovskites is their low stability when exposed to air and humidity.^48,49^ Various schemes have been used to shield perovskites from the environment such as adding a polymer layer or hBN encapsulation.^50–52^ In our structures, the cavity is directly fabricated on hBN and subsequently transferred on top of the perovskite. Moreover, we use a PMMA layer to index-match the surroundings of the cavity. Combining the hBN encapsulation with a polymer coating, we find that our structures become extremely stable. We did not observe any signs of degradation in our samples after shelf lifetimes of over one year at ambient conditions (see Supporting Information).

In a first step, we characterize the fabricated perovskite-cavity structures using optical transmission and photoluminescence (PL) spectroscopy. Figure 2a and b shows the angle-resolved transmission spectrum (dispersion relation) of an array before and after transfer, respectively. *k*_*y*_ denotes the in-plane wavenumber, which is related to the angle θ of transmitted light by *k*_*y*_ = (2*π/λ*) sin θ, with λ being the wavelength. For the bare array, the dispersion follows the typical shape of SLRs. Due to the coupling between the two diffracted orders of light, an avoided crossing opens a bandgap at *k*_*y*_ = 0.^15–17^ We fit the shape of the upper SLR branch using a well-established model (see Supporting Information), depicted as a red dotted line in Figure 2a. We observe a good match with the experimental data, confirming the validity of the model. In Figure 2b we observe a strong energy shift due to the large refractive index of 2D perovskites as well as a pronounced bending of the modes. Such avoided crossing behavior is typical for strongly coupled systems.^53,54^ In this case the SLRs couple with the exciton absorption transition of the perovskites (2.38 eV). To confirm that we are indeed in the strong coupling regime we use a classical coupled oscillator model to fit the two modes:^54,55^

1 $$\det\left(\begin{array}{cc} E_{\mathrm{SLR}}-i\gamma_{\mathrm{SLR}}+s & \Omega /2\\ \Omega /2 & E_{X}-i\gamma_{X} \end{array}\right)=0$$

with *E* and γ being the energy and line width of the two uncoupled modes, SLR and exciton (X), and Ω the Rabi splitting. Following a previously reported procedure,^54^ we include a shifting parameter *s* to take into account the strong shifting of the SLR mode due to refractive index change after transfer. The lower polariton branch derived from the model is shown as a red dotted line in Figure 2b and thoroughly reproduces the measured data. We determine a coupling strength of 144 meV, which is higher than the strong coupling limit of 72 meV given by the uncoupled mode and exciton line widths: (γ_X_ + γ_SLR_)/2. We observed strong coupling features in all fabricated samples (8 in total), with coupling strengths varying between 100 and 270 meV (see Supporting Information). For some samples the coupling strength even exceeded the ultrastrong coupling limit^56^ (Ω/*E* > 10%). Using a 405 nm continuous-wave laser to excite the samples, we see the same strong coupling features in PL, as shown in Figure 2c.

**Figure 2:**
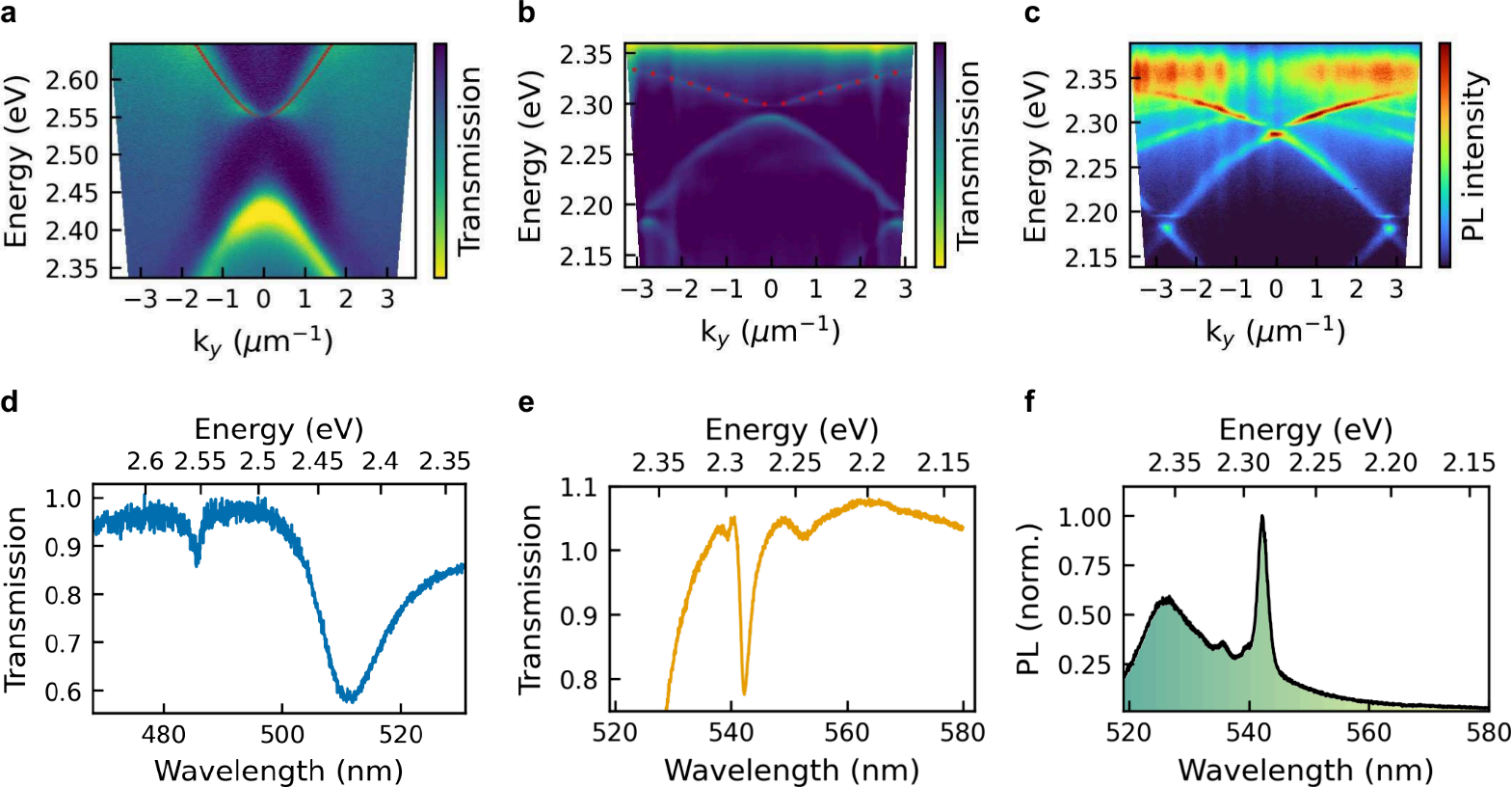
Optical measurements of 2D perovskites coupled to nanoparticle arrays. a) Angle-resolved transmission spectrum of an array on hBN and b) the same array after transfer on a 2D perovskite. c) Angle-resolved PL of the full stack. d) Crosscut of the angle-resolved transmission measurement at *k*_*y*_ = 0 for the bare array and e) the same array after transfer on a 2D perovskite. f) Crosscut of the angle-resolved PL of the full stack at *k*_*y*_ = 0.

The spectra in Figure 2d–f show the crosscut at *k*_*y*_ = 0 in the dispersion plots above and allow us to estimate the line width of the modes. Strikingly, we observe a pronounced narrowing of the mode line width from 72 to 9 meV (full width at half-maximum) after it is transferred on the 2D material stack. We observe the same line width narrowing effect in PL as well as a strong emission enhancement at the bandgap energy (see Figure 2f). Such a narrow line width for the polariton modes cannot be explained using the classical approach for strongly coupled oscillators, as it is far below both the emission line width of the perovskite and the bare cavity. We find that this effect can reduce the line width down to 5 meV for optimized structures corresponding to a quality factor close to 500 (see Supporting Information). We explore this phenomenon further in Figure 3, which summarizes the results of a total of 8 samples. Part of the line width narrowing most likely comes from the high refractive index active material inside the layered structures, that is, SLR modes can hybridize with waveguided slab modes.^57^ We investigated this scenario by simulating the effect of placing a high refractive index material close to a nanoparticle array. Figures 3a and b show the simulated dispersion of a bare cavity on hBN and with the additional hBN and 2D perovskite layer, respectively. This simulation does not account for any strong coupling or quantum effect for the shape and line width of the modes, but only on the refractive index change in the surroundings. This enables us to single out the expected impact of a SLR-waveguide mode hybridization.

**Figure 3:**
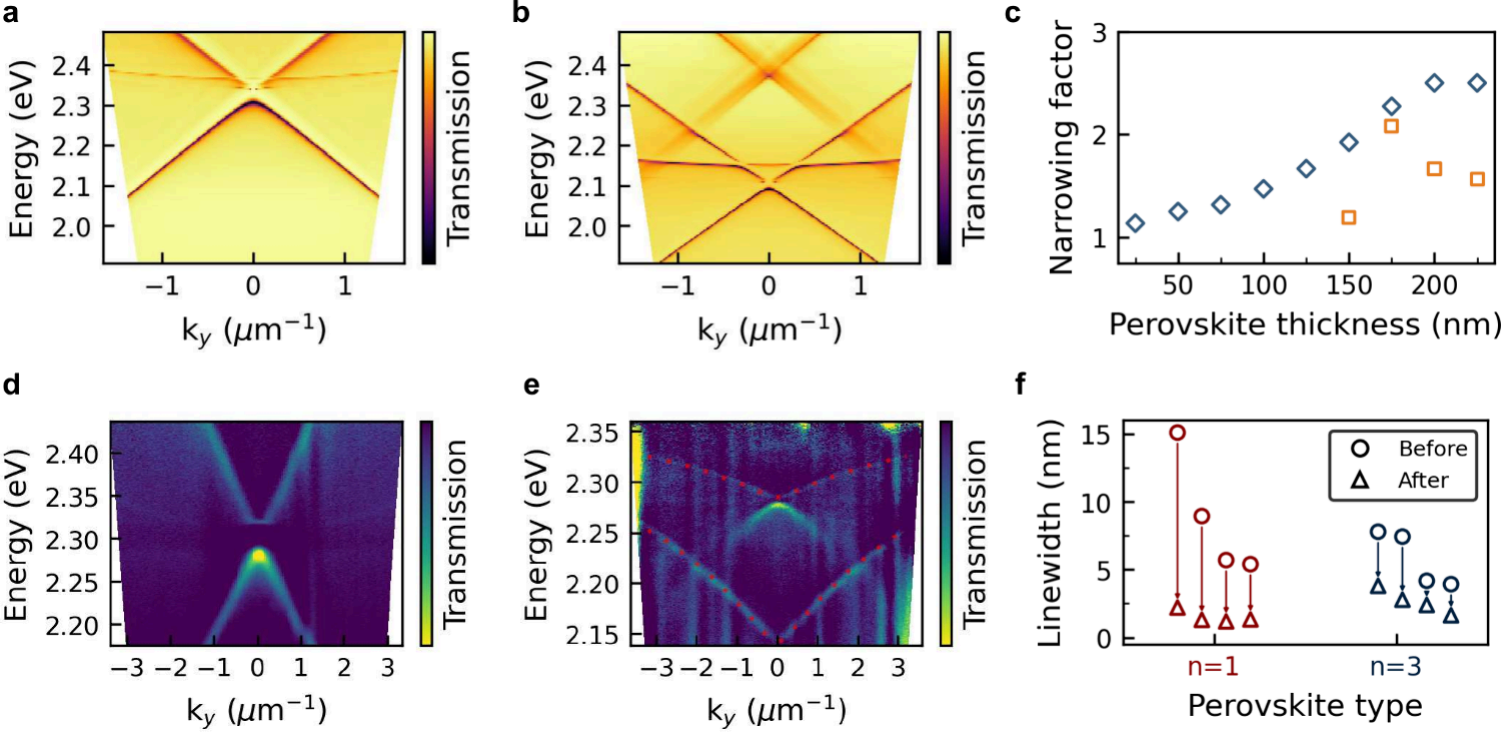
Ultrastrong coupling and line width narrowing. a) Simulation of the dispersion relation of a plasmonic nanoparticle array on a 30 nm thick hBN flake and b) on a 150 nm thick perovskite encapsulated by two hBN flakes with thicknesses 30 and 40 nm (bottom). c) Simulation of the line width narrowing factor of the TE modes as the perovskite thickness is increased. In blue (diamonds) the main mode and in orange (squares) a second mode that appears for thicker flakes. d) and e) Measured dispersion relation with the same geometries as the ones simulated in (a) and (b). The data in**e)** is normalized at each energy for better visibility of the modes. The polariton modes are fitted (red dotted line) using the procedure described above resulting in a coupling strength of 210 and 270 meV for the higher and lower energy modes, respectively. f) Measured line widths for multiple nanoparticle arrays before and after transferring on *n* = 1 and *n* = 3 2D perovskite layers. Arrows highlight the strong narrowing beyond the PL line width after transfer on the active material.

We find that the simulation predicts well the spectral location of the modes after transfer. As the thickness of the perovskite layer is increased, we notice the appearance of a second mode, which we also observed experimentally in Figure 3e for a second sample fabricated with a perovskite with a thickness above 150 nm. We observed the appearance of this second mode when using thicker perovskite layers, in accordance with the line width simulations shown in Figure 3c. Importantly, the simulation also shows a thickness-dependent line width narrowing from the waveguide coupling. Even so, we find that the line width narrowing factor predicted by the simulation is much lower than our experimental results for *n* = 1 perovskite (see Figure 3f). Interestingly, the line width narrowing is much smaller and in the range of the expectation for *n* = 3 perovskites. The latter consists of three inorganic layers separated by organic spacers, increasing the thickness of the natural quantum well. From a structural point of view, this only slightly changes the refractive index of the material. Optically, however, the emission shifts to lower energies and both the exciton binding energy and importantly the oscillator strength reduce strongly.^58^ This suggests that the light-matter coupling is responsible not only for the strong coupling of excitons to the cavity, but also for the line width narrowing of the hybrid modes.

The unusual line width narrowing can be attributed to effects associated with strong coupling, approaching the boundaries of ultrastrong coupling regime. Here, a single, homogeneously broadened excitonic resonance interacts with the cavity mode — rather than a collection of inhomogeneously broadened excitons — which can significantly reduce spectral broadening.^59^ The resulting line width can be estimated as half the sum of homogeneous line width and cavity line width.^60^ The inhomogeneous broadening stemming from phonons, static disorder and defects typically dominate at room temperature,^61^ resulting in a total line width of 70 meV with a purely homogeneous contribution of around 20 meV.^62^ Therefore, this effect could lead to a strong narrowing of the coupled mode line width. Additionally, motional narrowing may contribute to line width reduction in our system: the lower effective mass of exciton-polaritons compared to bare excitons facilitates enhanced delocalization, enabling an effective averaging over static disorder potentials.^63^ Overall, both effects address the large inhomogeneous broadening generally present in perovskites, enabling narrow line widths in our devices.

Our optical measurements and simulations provide solid evidence for ultrastrong coupling of perovskite excitons and plasmonic cavities. In a next step we demonstrate that these hybrid modes can be excited *electrically* via electron tunneling and subsequent energy transfer, as illustrated in Figure 4a. The tunnel junction consists of a graphene-hBN-graphene heterostructure that is placed adjacent to the perovskite layer. The energy lost by inelastic electron tunneling is transferred via energy transfer to perovskite excitons.^31,64^ We note that the tunnel junction and hence the electrical pathway are separated from the perovskite-cavity system, that is, there is no direct charge injection into the active material. This excitation mechanism has important advantages over direct charge injection schemes.

**Figure 4:**
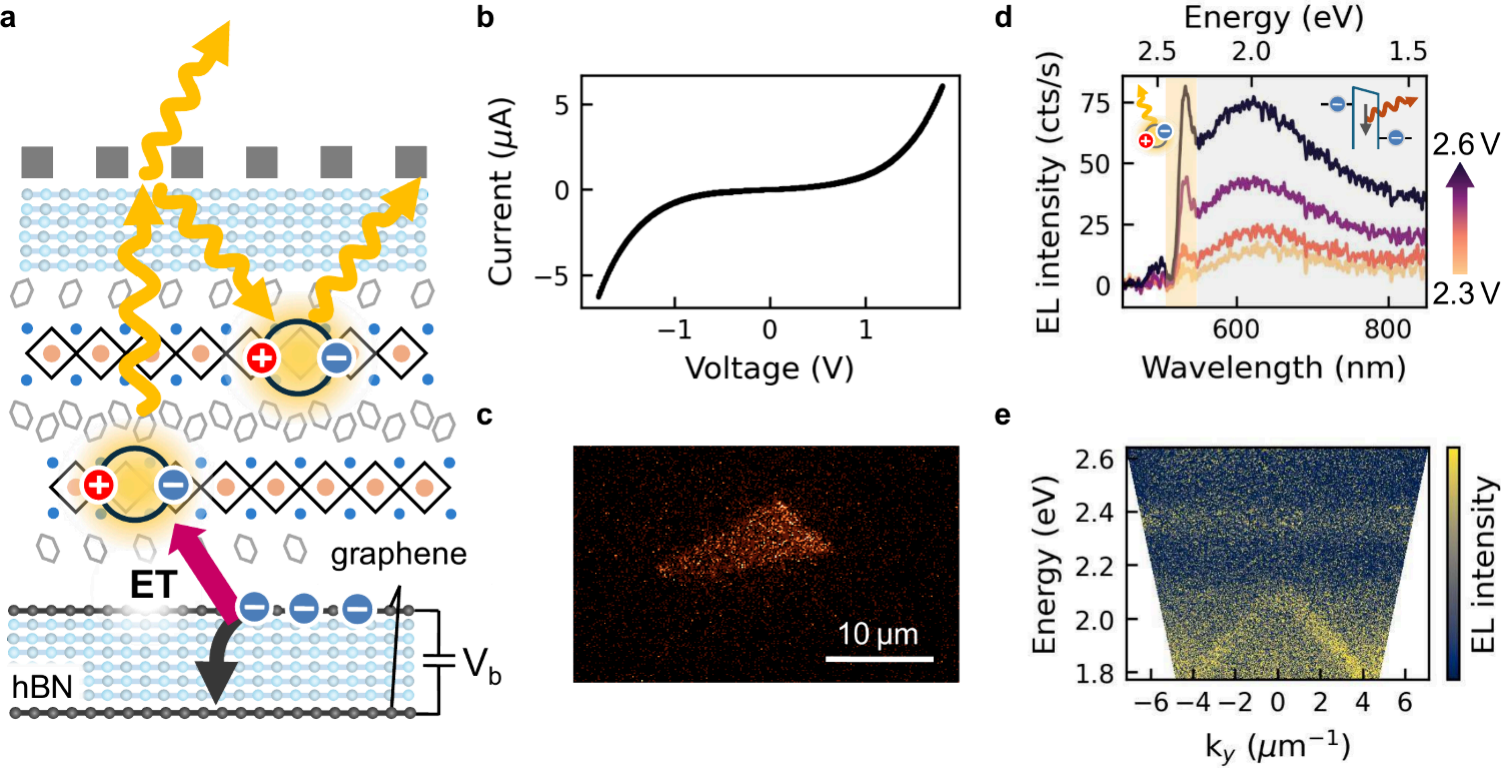
Electrically excited 2D perovskite coupled to a nanoparticle array cavity. a) Schematic of the device structure, with the same structure as before placed directly on top of a graphene-hBN-graphene tunnel junction. b) Tunneling current as a function of applied voltage. c) Spatially resolved electrical emission from the device. The triangular outline matches the graphene-grapehen overlap area. d) Spectrally resolved electroluminescence (EL) intensity of 2D perovskite without cavity for increasing bias voltage. Inset depicts the emission from excitons (yellow shaded area) and tunneling emission by directly coupling to photons (gray shaded area). e) Angle-resolved EL of the electrically excited nanoparticle array-2D perovskite sample, with the SLR mode clearly visible at lower energy and the exciton band above.

First, no electric field is applied to the perovskite, which greatly enhances the stability of the device. Electric field-induced degradation, such as ion migration and Pb reduction, can be mitigated.^65^ Second, no direct contact from a top electrode to the active material is needed, which allows us to directly stack the nanoparticle array on top. Third, as excitons are directly generated via energy transfer, there is no imbalance of the injected electron- and hole density, enhancing efficiency and stability. To build the device, a van der Waals tunneling diode is fabricated from graphene layers and a thin hBN barrier (ca. 2 nm), and the perovskite layer and the transferable cavity are subsequently added.

Figure 4b shows the resulting voltage-dependent current, exhibiting a typical tunneling shape. The atomically flat nature of graphene and hBN leads to a highly homogeneous emission only from the overlapping area of the two graphenes, as depicted in Figure 4c. Without the nanoparticle array, we observe a sharp emission from the excitons in PEA_2_PbI_4_ at around 530 nm, as can be seen in Figure 4d. The emission becomes stronger with increasing voltage. The additional broad emission originates from the direct coupling of inelastically tunneling electrons to photons, as further depicted in the inset of Figure 4d.^66^ While we observe stable emission for more than 1 h of measurement at ambient conditions in air, the overall external quantum efficiency, e.g. creating a photon from the perovskite emission per electron, is only around 10^–7^. This low electron-to-photon conversion is generally in the range of such energy transfer tunneling junctions and further lowered by the high energy of the exciton at around 2.36 eV.^31,67^ Here, a large fraction of tunneling electrons couples directly to light and thus reduces the efficiency of the exciton excitation, as shown by the gray shaded emission in Figure 4d. Additionally, the organic spacer further increases the distance between the tunneling dipole inside the junction and the active, inorganic quantum wells in the perovskite. Nevertheless, due to the additional losses inside the perovskite, we estimate 2 orders of magnitude higher efficiency of the energy transfer compared to the direct coupling of light. In Figure 4e we show the angle-resolved electrical emission spectrum of the full device. The nondispersive emission at 2.36 eV is attributed to the exciton emission and the Λ-shaped feature between 1.8 and 2.1 eV matches the dispersion of the cavity mode. Again, most of this emission is connected to guiding the broad tunneling emission, which dominates at this wavelength.

In this work, we demonstrate the integration of plasmonic nanoparticle arrays into 2D material stacks. Using 2D organic–inorganic perovskites, we measure strong and even ultrastrong coupling between the exciton and the cavity modes of our structure, as well as strong enhancement of the emission. We also observe a pronounced line width narrowing of the cavity modes after transfer, which we attribute partly to a wave-guiding effect and partly to the interaction of the cavity with the exciton resonance beyond the usual strong-coupling regime. This results in polariton line width as narrow as 4.8 meV (1.2 nm). Furthermore, we show that we can use an energy transfer method to electrically excite 2D perovskites, eliminating the need for conventional sandwiched electrode architectures. We show that the electroluminescence of the 2D perovskite couples to the cavity modes of the plasmonic array. We do not see clear evidence for the strong coupling using electrical injection, but the structure shows promising electroluminescence from both the cavity modes and the perovskite. Notably, the electrically driven devices maintain emission under ambient conditions for over an hour. However, the efficiency and stability still needs improvement by engineering the contact area between the perovskite and graphene and optimizing the chemical composition of the perovskite. With recent advances in the chemical composition of 2D perovskites for increased gain^68^ we believe that this work will contribute toward novel optoelectronic functionalities on the nanoscale.

## Materials and Methods

### Perovskite Crystal Synthesis

Lead­(II) iodide (99%) and phenethylamine (≥99%) were purchased from Sigma-Aldrich. Lead­(II) oxide (99.9%) was purchased from Alfa Aesar. Hydriodic acid (57% aqueous solution, stabilized with 1.5% hypophosphorous acid) was purchased from ABCR. Methylammonium iodide (99.99%) was purchased from Greatcell Solar Materials. All chemicals were used as received without further purification.

#### Synthesis of Phenethylammonium Iodide (PEA)­I

Thirteen milliliters of hydriodic acid was added dropwise to a mixture of 10 mL of phenethylamine and 20 mL of ethanol (anhydrous) with stirring; the mixture was cooled with an ice bath. The mixture was kept stirring for 1 h. The resulting solution was evaporated in a rotary evaporator at 50 °C until complete removal of liquid. The obtained solid was washed with diethyl ether three times and recrystallized from ethanol with diethyl ether. The final product was obtained after drying under vacuum at 50 °C overnight (14 g).

#### Synthesis of Single Crystals of (MA)_*n*−1_(PEA)_2_Pb_*n*_I_3*n*+1_ (*n* = 1, 3)

Single crystals were grown using a previously reported cooling method. Briefly, precursors containing lead oxide, methylammonium iodide, and phenethylamine with specific ratios were dissolved in hydriodic acid (HI) solution (57% w/w in water) at ∼110 °C and kept at this temperature for 4 h. The solutions were then slowly cooled to room temperature at a rate of 1 °C/h. The ratios were 1.72/0/3.45 mmol, and 10/24/1 mmol for *n* = 1,3, respectively, in 30 mL of HI solution. The obtained crystals were filtered under vacuum and dried with diethyl ether.^69^

### Cavity Fabrication

hBN crystals (National Institute for Materials Science, Japan) were exfoliated on a polydimethylsiloxane (PDMS) film (Gel-Pak). Thin flakes of around 30 nm were selected by contrast and the thickness was confirmed using atomic force microscope (AFM) measurements. The hBN flakes were directly transferred from the PDMS stamps to a borosilicate glass substrate. The transfer was performed by contacting the hBN flake on the glass at low angles and slowly retracting the PDMS stamp while heating at 40 °C to increase hBN-glass adhesion. Square arrays of Al nanoparticles were subsequently fabricated on hBN flakes using EBL and electron-beam evaporation. The substrate was treated with O_2_ plasma and spin-coated with a 200 nm of 950 K PMMA resist. After patterning the arrays, the PMMA was developed in a 1:3 ratio of deionized water to isopropanol solution. A 25 nm layer of Al was evaporated onto the patterned PMMA at 5 nm s^–1^ and <5 × 10^–8^ mbar to limit the oxidization of the nanoparticles during deposition. This led to cylindrically shaped nanoparticles with diameters between 65 and 75 nm. The lateral dimensions of the arrays were chosen according to the size of the exfoliated hBN between 50 and 100 μm. The samples were left for 16 h in acetone for lift-off.

### 2D Material Stacking

For electrically excited samples, graphene (NGS, Naturgraphit GmbH, Germany) and hBN (National Institute for Materials Science, Japan) were exfoliated from bulk crystals onto 100 nm thick SiO_2_/Si substrates. The graphene flakes were chosen by optical contrast. The hBN flake were chosen by optical contrast and their thickness was confirmed using AFM measurements. The graphene-hBN-graphene stack were subsequently picked up using a polymer-based dry pick-up and transfer method.^70^ The layers were transferred onto a glass substrate and the polymer residues were removed in chloroform. 2D perovskites (phenylethylammonium-lead iodide, PEA_2_PbI_4_), (Functional Inorganic Materials, ETH) were exfoliated and transferred onto the tunnel junction using the same PDMS transfer method described in the previous subsection. Finally, the hBN-cavity was picked up using a low temperature polymer-based dry pick-up and transfer method^71^ in a controlled Ar environment. This method based on poly­(propylene)­carbonate (PPC) films has the advantage of requiring low temperatures for the transfer process (40 °C for pick-up and 70 °C for transfer). This was done to limit the thermal degradation of the 2D perovskite layers. In a final step, 11% anisole/PMMA was dropcasted on the structures for index-matching purposes.

### Optical Measurements

Optical measurements were performed using a Nikon TE300 inverted microscope and measured under ambient conditions in air. Dispersion relations were obtained from white-light transmission measurements. A broadband halogen lamp was focused onto the sample using a low magnification objective (Olympus, 10x, 0.3NA) from the top side. The transmitted light was collected by another objective (Nikon, 50x, 0.8NA). Angle-resolved data was obtained by focusing the back focal plane onto the entrance slit of a spectrometer (Princeton Instruments Acton SpectraPro 300i) equipped with a charge-coupled device (CCD) camera (Princeton Instruments BLAZE 400). Energy and *k*_*y*_ values were obtained using the same approach as in our previous works.^44^ PL measurements were obtained by exciting and collecting light through the same objective (Nikon, 50×, 0.8NA). A long-pass filter was used to separate the 405 nm continuous-wave laser pump from the sample emission, before focusing the back focal plane of the emission on the detector as described above. Electrical excitation of the samples was realized by connecting the sample to a direct-current voltage source (Keithley Instruments 2602B) and varying the applied gate voltage. The electroluminescence was collected following the same procedure as the transmission and PL measurements. The efficiency of the EL is estimated from the known transmission function of the setup and the simultaneously measured current.

## Numerical Simulations

Numerical simulations were conducted using COMSOL Multiphysics 6.2 within the Wave Optics module. The optical response of the nanoparticle array was modeled using a single-particle unit cell, with Floquet-periodic boundary conditions applied in the lateral directions (*x* and *y*), and perfectly matched layers (PMLs) implemented along the vertical (*z*) axis. The refractive indices of glass, PMMA, hBN, and perovskite were 1.52, 1.49, 2, and 1.76, respectively. The optical constants for crystalline Al were adopted from tabulated values.^72^ To calculate the transmission spectrum of the samples, the system was excited by a plane wave at varying incident angle. The nanoparticle was modeled as a cylinder with a diameter of 65 nm and a height of 25 nm, and the period in *x* and *y* was 345 nm. The incident electric field was set to be *y*-polarized.

## Supplementary Material



## Notes

Further details regarding experimental and fitting procedures, data analysis, and additional experimental results (PDF)
