# Supplementary material for: Transferable Plasmonic Arrays Enabling Strong Coupling with Layered Perovskites in an Active Diode Architecture
Source: Nano Lett. 2025 Dec 31;26(5):1616–24. doi: 10.1021/acs.nanolett.5c04569 (PMC12904071; doi:10.1021/acs.nanolett.5c04569)
Supplement: Supplementary file 1 [file nl5c04569_si_001.pdf]

Supporting information

Transferable Plasmonic Arrays Enabling Strong Coupling with Layered Perovskites in an Active Diode Architecture

Fabien Dorey[a],[†] Jonas D. Ziegler[a],[†] Antti J. Moilanen,[†,⊥] Oleh Hordiichuk,[‡,#] Gabriel Nagamine,[¶] Takashi Taniguchi,[§] Kenji Watanabe,[‖] David J. Norris,[¶] Maksym V. Kovalenko,[‡,#] Gabriele Rainò,[‡,#] and Lukas Novotny[*,†]

†*Photonics Laboratory, ETH Zürich, CH-8093 Zürich, Switzerland*

‡*Laboratory of Inorganic Chemistry, Department of Chemistry and Applied Biosciences, Institute of Inorganic Chemistry, ETH Zürich, CH-8093 Zürich, Switzerland*

¶*Optical Materials Engineering Laboratory, Department of Mechanical and Process Engineering, ETH Zürich, CH-8092 Zürich, Switzerland*

§*International Center for Materials Nanoarchitectonics, National Institute for Materials Science, Tsukuba, Ibaraki 305-004, Japan*

‖*Research Center for Functional Materials, National Institute for Materials Science, Tsukuba, Ibaraki 305-004, Japan*

⊥*Department of Physics and Mathematics, Faculty of Science, Forestry and Technology, University of eastern Finland, Joensuu, FI-80101, Finland*

#*Laboratory for Thin Films and Photovoltaics, Empa - Swiss Federal Laboratories for Materials Science and Technology, CH-8600 Dübendorf, Switzerland*

E-mail: zieglerj@ethz.ch

---

[a]equal contribution

Optical properties of 2D perovskites

2D perovskites were synthesized as described in the methods section of the main text. By varying the number of perovskite layers (1 or 3) between two ligand layers, the exciton confinement and, therefore, the emission energy can be tuned, as seen in Figure 1.

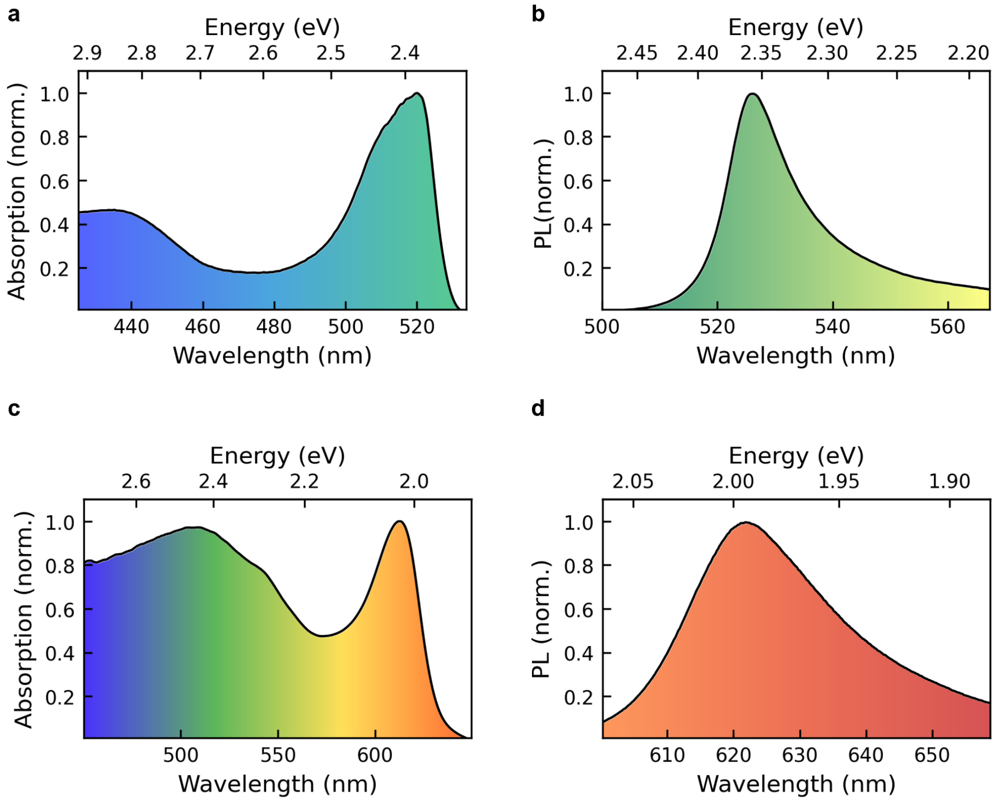

Figure 1: Absorption **(a,c)** and emission **(b,d)** of exfolliated n=1 (a,b) and n=3 (c,d) 2 Dimensional perovskite. The plots are normalized to the maximal values measured in the data range.

Aluminum nanoparticle plasmonic arrays.

Aluminum arrays were fabricated on hBN using the process described in the materials section of the main text. In particular, high deposition speeds of the aluminum ($5\,\mathrm{nm\,s^{-1}}$) were critical to reach high quality factor values (up to 100 and 160 for n=1 and n=3 respectively). We modeled the resonances using a Fano function due to the asymmetry of nanoparticle array resonances.

$$f(x) = y_0 - a\,\frac{\left(q + \frac{x-x_0}{\gamma}\right)^2}{(x-x_0)^2+\gamma^2}$$

With y_0 the baseline offset, a the dip amplitude, x_0 the resonance position, γ the width parameter, and q the Fano asymmetry parameter. The FWHM was determined by taking the fitted width at the half maximum transmission value, which was taken directly from the experimental values. Note that cavities from earlier fabrication rounds, which were not yet optimised, were used in the main text as well as in this supporting information, as they exhibited the same physical phenomena. To fit the uncoupled array modes we used the following formula:

$$E_{SLR} = a \cdot \sqrt{bk^2 + c} + d$$

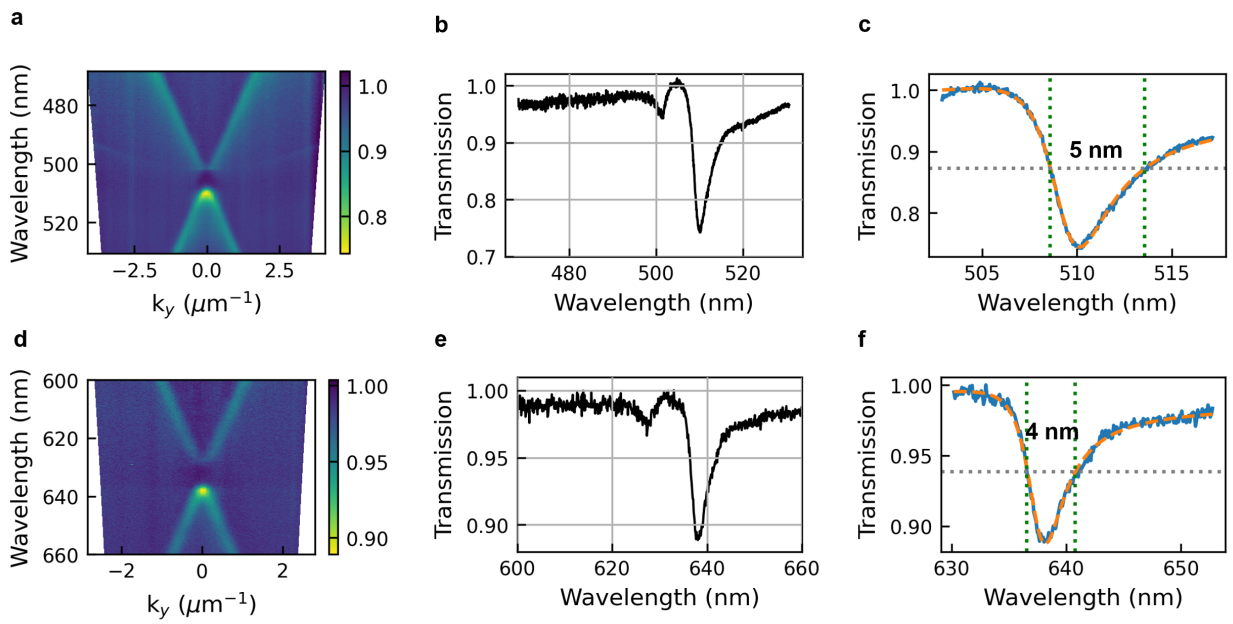

Figure 2: White light transmission of plasmonic arrays on hBN. (a-c) n=1: **a)** Angle resolved transmission, **b)** Crosscut of transmission through the bandgap at $k_y = 0$. **c)** Region used for the fitting of the resonance linewidth. , **(d-f)** n=3: Transmission is represented analogously to the cavities prepared for n=1 samples.

Angle-resolved transmission of further samples.

All 2D perovskite-nanoparticle array samples showed avoided crossing features of their modes with the exciton absorption energy of the 2D perovskite. Figure 3 shows the transmission of four different samples: Two were fabricated using n=1 samples (a,b) and two with n=3 samples (c,d). Using the model described in the main text we find that all four samples are above the strong coupling limit with coupling strength of 137 meV and 178 meV for the n=1 samples and 101 meV and 141 meV for the n=3 samples. For optimized structures linewidth down to 4.5 meV (1.18 nm) and 7.1 meV (2.4 nm) were measured for n=1 and n=3 samples, respectively. Table 1 shows the linewidth measured on the various samples before and after transferring the cavities on the perovskite layer.

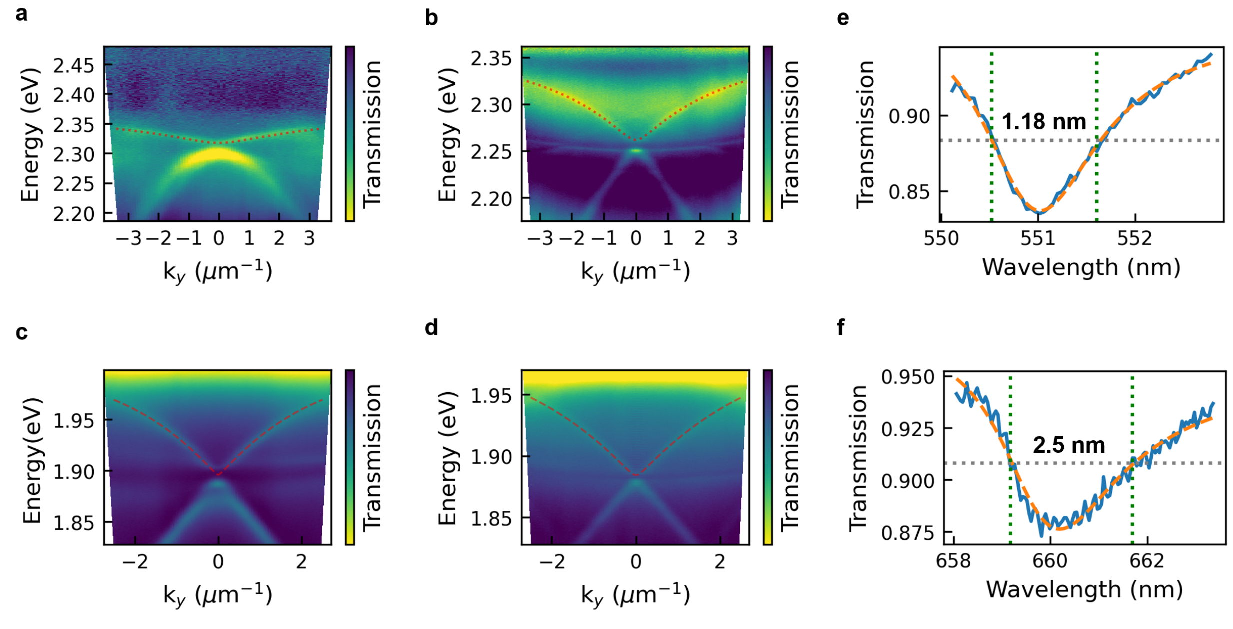

Figure 3: Angle-resolved transmission of n=1 **(a, b)** and n=3 **(c, d)** 2D perovskites coupled to a nanoparticle array. The polariton resulting from the fitting of the experimental data is shown as red dotted lines. **(e, f)** Transmission crosscut at the standing wave mode shown in (b) and (d), respectively.

Table 1: Absolute linewidth and narrowing factor measured with n=1 2D perovskites

Bare array linewidth (nm)	29.87	15.08	8.93	5.69	5.38
Full stack linewidth (nm)	3.90	2.2	1.3	1.18	1.33
Narrowing factor	7.66	6.86	6.87	4.82	4.05

Table 2: Absolute linewidth and narrowing factor measured with n=3 2D perovskites

Bare array linewidth (nm)	7.78	7.42	4.17	3.92
Full stack linewidth (nm)	3.8	2.78	2.39	2.4
Narrowing factor	2.04	2.67	1.74	1.63

Sample stability

The samples were found to be very stable due to the combination of a protective polymer layer and hBN encapsulation. No sign of degradation of the perovskites was observed optically (see Figure 4) or from their emission profile (see Figure 5a and b). We also measured the angle-resolved photoluminescence of two different samples after one year in Figure 5. Again, we observe a very similar response after one year of shelf lifetime in ambient conditions, showing that the nanoparticle arrays as well do not degrade or oxidize within that timeframe when covered by a protective polymer layer.

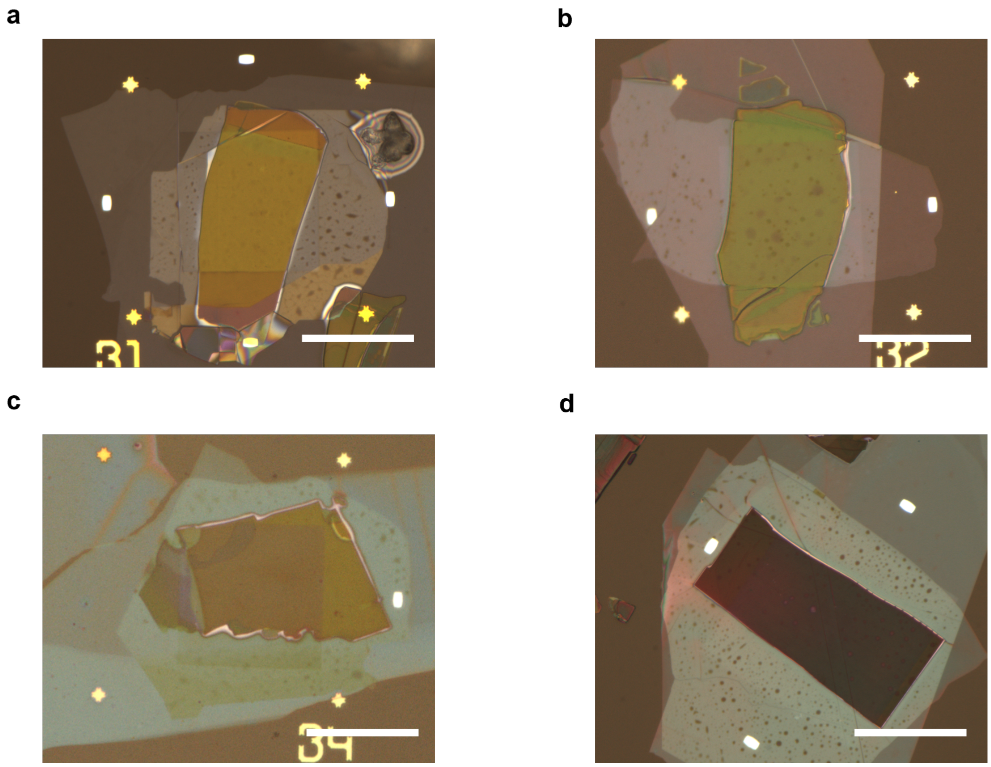

Figure 4: Optical microscope image of various n=1 samples **(a-c)** and one n=3 sample **d)** over one year after fabrication. (Scale bar: 25 µm)

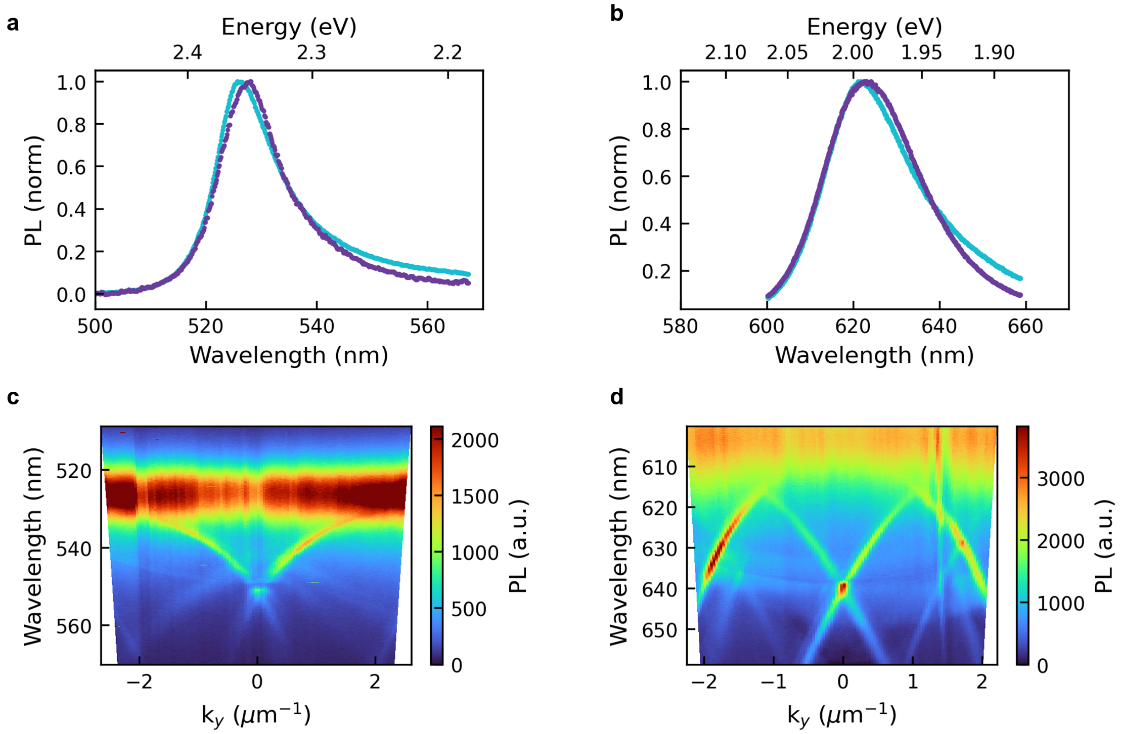

Figure 5: Photoluminescence of two samples one year after fabrication. **a, b)** In blue the emission measured shortly after fabrication and in violet the emission measured over one year after fabrication. Note that the emission was measured next to the array, so that the emission profile would not be affected by it. Below in **c)** and **d)** the angle-resolved emission profile of the same sample measured in (a) and (b), respectively.
